# STARD—rapid screening for the 6 most common *G6PD* gene mutations in the Chinese population using the amplification refractory mutation system combined with melting curve analysis

**DOI:** 10.1097/MD.0000000000010426

**Published:** 2018-04-27

**Authors:** Zuqian Fan, Xunjin Weng, Guosheng Huang, Zhijian Pan, Zhao Long, Qiongying Fan, Weijun Tang, Lin Fang, Ju Long, Tian Hu, Yongxia Huang, Lei Sun

**Affiliations:** a Department of Clinical Laboratory, Qinzhou Maternal and Child Health Hospital; b Qinzhou Key Laboratory of Molecular and Cell Biology on Endemic Diseases, Qinzhou; c Laboratory of Medical Genetics, Qinzhou Maternal and Child Health Hospital, Guangxi, PR China.

**Keywords:** DNA mutational analysis, glucosephosphate dehydrogenase deficiency, neonatal screening, point mutation, transition temperature

## Abstract

Dot-blot hybridization and high-resolution melting curve methods are used to detect *G6PD* gene mutations; however, the performance and throughput limitations of these methods hinder their use for screening large populations. For simple screening, we developed a novel approach called “Amplification Refractory Mutation System combined with Melting Curve Analysis (ARMS-MC),” which enables rapid and batch-based detection of the 6 most common *G6PD* mutations.

In this method, we established 4 PCR reaction systems that can be used to detect the 6 most common *G6PD* mutations (c.95A>G, c.392G>T, c.871G>A, c.1024C>T, c.1376G>T, and c.1388G>A) in the Chinese population.

The ARMS-MC method was evaluated with 174 cases of clinical G6PD-deficient samples, and the results were verified by direct sequencing at *G6PD* gene exons. The results showed that 170 samples had ≥1 of the 6 mutations, which accounted for 97.70% of all mutations. These results were consistent with the results of direct sequencing with 100% accuracy and specificity. Sequencing validation revealed other mutations in the 4 samples in which no mutation was detected by the ARMS-MC method.

ARMS-MC provides a rapid, simple, inexpensive, and accurate screening method for detecting the most common *G6PD* mutations in Chinese people.

## 1 Introduction

As one of the most common enzyme deficiency diseases in human beings, the X-linked recessive disorder glucose-6-phosphate dehydrogenase (G6PD) deficiency occurs frequently in tropical and subtropical regions as well as in southern China, with approximately 400 million people affected worldwide.^[1,2]^ The clinical manifestations of favism vary considerably from no symptoms to neonatal jaundice, acute hemolysis caused by drugs or infection, and severe chronic nonspherical cell hemolytic anemia. In serious cases, severe kernicterus may occur in the neonatal period, resulting in death or permanent nervous system damage.^[3]^ Early diagnosis and prevention of the disease is an important part of prenatal and postnatal care in China. Disease prevention focuses on detection of the presence of G6PD deficiency, providing reasonable advice to patients, avoiding the use of drugs that may cause hemolysis, and appropriate treatment of the resulting neonatal hyperbilirubinemia.

Quantifying G6PD enzyme activity is the main experimental method currently being used for clinical diagnosis of G6PD deficiency.^[4]^ However, different types of mutations lead to different phenotypes; in addition, the existing enzyme activity analysis methods cannot effectively detect female carriers of *G6PD* gene mutations or accurately determine whether individuals with clinical symptoms of acute hemolysis have G6PD deficiency. Therefore, the DNA detection method has important applications for clinical and differential diagnosis of G6PD deficiency, genetic counseling, and prenatal and postnatal care.^[5]^

Currently, the frequently used molecular methods for rapid diagnosis of *G6PD* mutations include denaturing gradient gel electrophoresis,^[6]^ the amplification refractory mutation system (ARMS),^[7]^ probe melting curve (MC) analysis,^[8]^ microarray,^[9]^ denaturing high-performance liquid phase chromatography (HLPC),^[10]^ matrix-assisted laser desorption/ionization time-of-flight mass spectrometry (MALDI-TOF MS),^[11]^ PCR dot-blot hybridization, and high-resolution melting (HRM).^[12,13]^ These methods are effective for detecting mutations. However, these methods also have some disadvantages such as costly, cumbersome, time consuming, low throughput, or complex operation. Therefore, they are not suitable for large-scale mutation screening or epidemiological studies.

In China, ARMS, HRM, and dot-blot hybridization are the main detection methods for G6PD mutations. The cost and requirements of ARMS and HRM are relatively low. However, due to the limitations of the technical conditions these 2 methods use, a single reaction can be used to evaluate only 1 mutation locus. Therefore, single-locus detection is rapid and simple, but multitube operation is usually required for multilocus detection; this limitation seriously affects the ability to conduct batch testing. ^[7,13]^ The PCR dot-blot hybridization technique has the advantage of multiloci detection. However, this method requires a membrane hybridization detection step after PCR. Application of this method to large-scale clinical testing can easily lead to contamination of laboratory DNA amplification products, affecting the accuracy of the experiment.^[12]^

To establish a simple, fast, and multiloci detection method, we combined the ARMS and MC techniques. This combination allowed us to achieve simultaneous detection of the 6 most common *G6PD* mutations in China. A total of 174 samples from patients with known G6PD deficiency were tested using this method, and the frequency of each mutation was determined.

## 2 Materials and methods

### 2.1 Ethics statement

This study was conducted according to the principles expressed in the Declaration of Helsinki. The protocol for this study was approved by the Research Ethics Committee of Qinzhou Maternal and Child Health Hospital. Written informed consent was obtained from each participant for the collection of samples and for subsequent analyses.

### 2.2 Materials

Ten samples of healthy individuals validated by sequencing and 12 samples with mutations (1 male and 1 female sample for each of the c.95A>G, c.392G>T, c.871G>A, c.1024C>T, c.1376G>T, and c.1388G>A mutations) were used in the validation experiments for normal and mutant primers. A total of 174 clinical neonatal G6PD-deficient samples that were positive in enzyme activity testing (96 males and 78 females) were used to determine the accuracy and specificity of the method. The results for all clinical samples were verified by direct sequencing at *G6PD* gene exons.

### 2.3 Genomic DNA extraction

The Peripheral Blood Genomic DNA Extraction Kit produced by Tiangen Biotech Co., Ltd. was used to extract genomic DNA from peripheral blood. The concentration and quality of the extracted genomic DNA were measured and evaluated by Nanodrop, and the extracted genomic DNA was stored at −20°C.

### 2.4 Primer design and validation

Primers were designed to detect normal and mutated alleles at *G6PD* gene loci: 95, 392, 871, 1024, 1376, and 1388.

To verify the amplification primers for normal and mutated alleles at these loci, PCR amplification and agarose gel electrophoresis were performed on the 10 normal samples and 12 mutation samples. These samples were verified by sequencing using the normal and mutation detection primers that were designed. At the same time, the melting point of each locus was determined using the melting curve method to select primer combinations with different melting points.

### 2.5 PCR amplification and melting curve analysis

PCR amplification and melting curve analysis were performed using the CFX96 Real-Time System instrument produced by Bio-Rad. The amplification samples were 25 μL, and the reaction system consisted of 1×GoldStar Taq MasterMix (CWBIO) and 10 ng genomic DNA. Primer concentrations are shown in Table 1. The parameters were set as follows: predenaturation at 94°C for 3 minutes; 30 cycles of denaturation at 94°C for 30 seconds, annealing at 60°C for 30 seconds, and extension at 72°C for 30 seconds; and a final extension at 72°C for 10 minutes. Next, the temperature was slowly brought up to 90°C at a rate of 0.1°C/s. During this process, the fluorescence signal change was automatically monitored, and the software automatically performed the melting curve analysis.

**Table 1:**
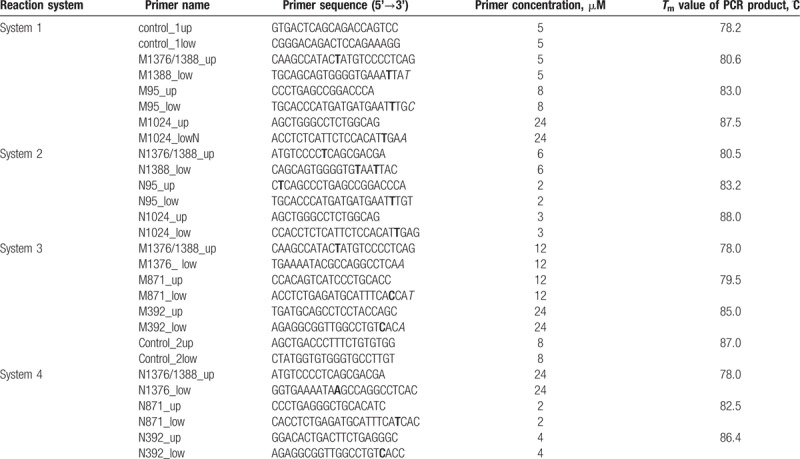
Detection primers for normal and mutation loci.

### 2.6 Detection of clinical samples

The 174 clinical G6PD-deficient samples were tested using the ARMS-MC method. All of the samples were evaluated using the mutant primer detection system (systems 1 and 3) and the normal primer detection system (systems 2 and 4) for hemizygous, homozygous, or heterozygous mutations. The frequency of each mutation was determined.

At the same time, sequencing analysis of the *G6PD* mutant loci was conducted for all of the samples (carried out by Gen Script Biotechnology Co., Ltd.) to verify the accuracy and specificity of the method.

## 3 Results

### 3.1 Design, validation, and combination of primers

**Figure 1:**
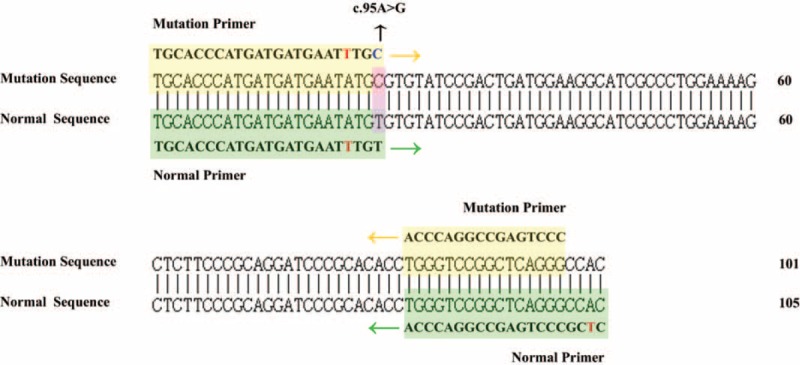
Principles of the design of ARMS primers. The 2 sequences in the picture are the normal sequence and the mutation sequence. The red base in the primer sequence is artificially introduced, and the blue base is the c.95A>G mutation locus.

In this study, 4 reaction systems including 14 pairs of primers were designed. Reaction systems 2 and 4 were used to detect normal loci, whereas reaction systems 1 and 3 were used to detect mutations. In addition to the mutation loci primers, each system contained a pair of primers named control 1 and control 2, which were used as endogenous controls to determine whether the PCR reaction system contained DNA. The specific primer sequences are shown in Table 1. The principles of ARMS primer design are shown using *G6PD* gene locus 95 as an example (Fig. 1).

### 3.2 Results of the melting curve analysis

**Figure 2:**
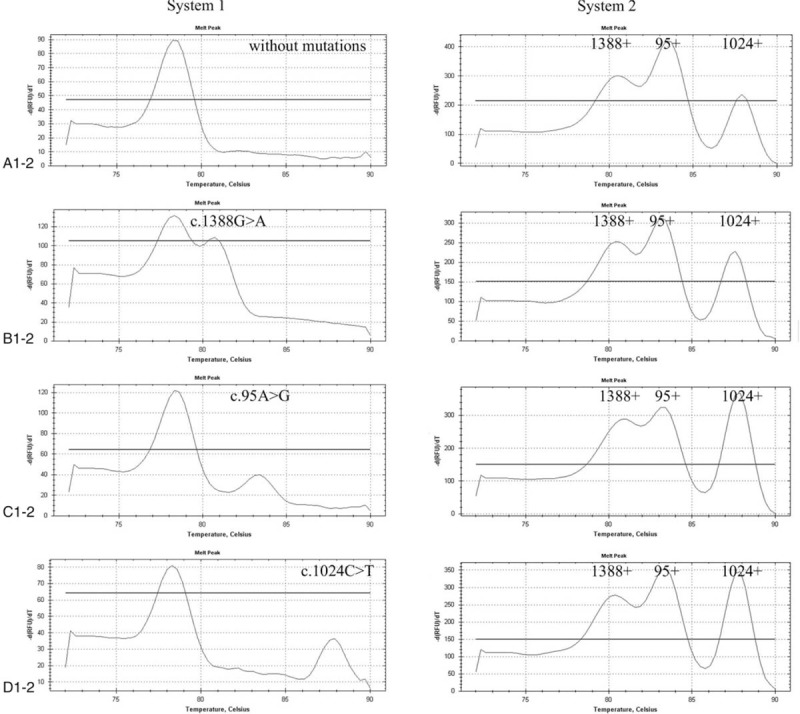
Melting curve analysis of heterozygous loci 1388, 95, and 1024. The samples were tested with systems 1 and 2 at the same time. (A) The results for an individual without the c.1388G>A, c.95A>G, and c.1024C>T mutations. (B–D) The results for a heterozygous individual with c.1388G>A, c.95A>G, and c.1024C>T mutations, respectively.

Reaction systems 1 and 2 were mainly used to identify mutations at loci 1388, 95, and 1024. Detection system 1 was mainly used to determine whether there were mutations at loci 1388, 95, and 1024. The peak at 78.2°C was the control peak of system 1 and was used to determine whether a DNA template was added during PCR amplification (Fig. 2, a-1). The melting points of the products at mutation loci 1388, 95, and 1024 were 80.6°C, 83.0°C, and 87.5°C, respectively (Fig. 2, b-1, c-1, d-1). The presence of a product peak at the corresponding melting point indicates the presence of a corresponding mutation. Detection system 2 was mainly used to determine whether mutations at loci 1388, 95, and 1024 were homozygous, heterozygous, or hemizygous, with melting points of 80.5°C, 83.2°C, and 88.0°C, respectively (Fig. 2, a-2, b-2, c-2, d-2).

**Figure 3:**
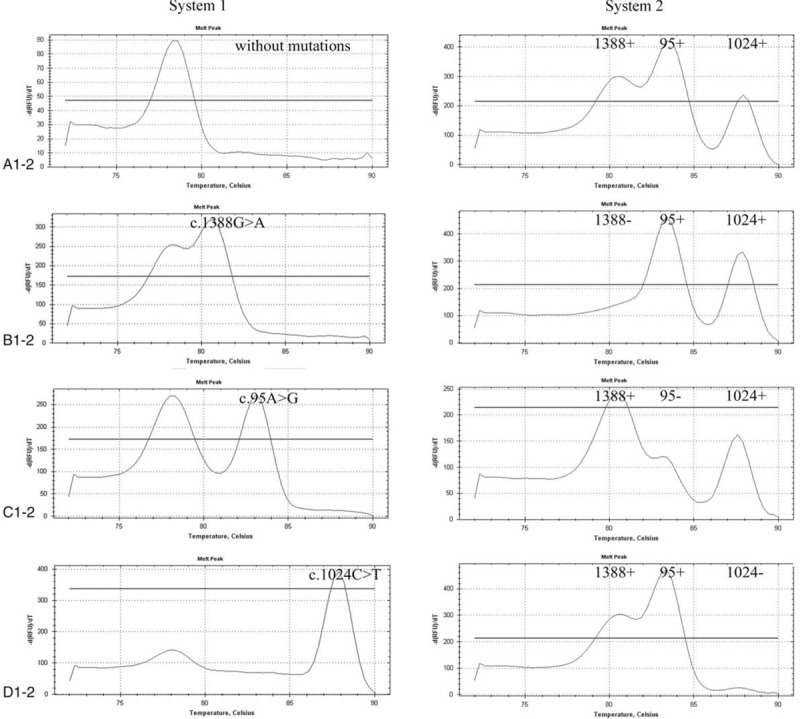
Melting curve analysis of heterozygous loci 1376, 871, and 392. The samples were tested with systems 3 and 4 at the same time. (A) The results for an individual without c.1376G>T, c.871G>A, and c.392G>T mutation. (B–D) The results for a heterozygous individual with c.1376G>T, c.871G>A, and c.392G>T mutation, respectively.

Reaction systems 3 and 4 were mainly used to identify mutations at loci 1376, 871, and 392. Detection system 3 was mainly used to determine whether there were mutations at loci 1376, 871, and 392. The peak at 87.0°C was the control peak of system 3 and was used to determine whether a DNA template was added during PCR amplification (Fig. 3, a-1). The melting points of the products at mutation loci 1376, 871, and 392 were 78.0°C, 79.5°C, and 85.0°C, respectively (Fig. 3, b-1, c-1, d-1). The presence of a product peak at the corresponding melting point indicates the presence of a corresponding mutation. Detection system 4 was mainly used to determine whether the mutations at loci 1376, 871, and 392 were homozygous, heterozygous, or hemizygous, with melting points of 78.0°C, 82.5°C, and 86.4°C, respectively (Fig. 3, a-2, b-2, c-2, d-2).

### 3.3 Melting curve analysis and sequencing of clinical samples

**Figure 4:**
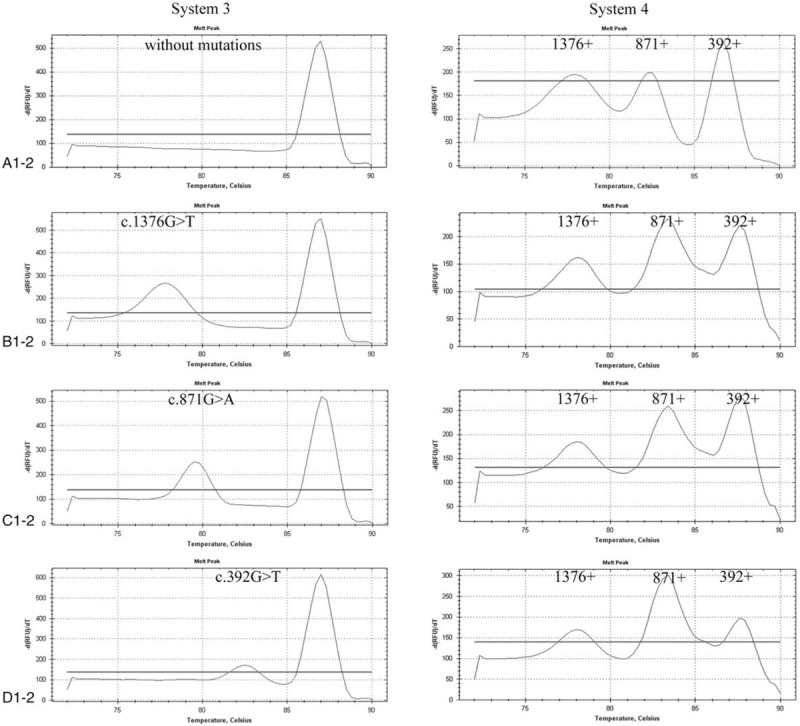
Melting curve analysis of homozygous or hemizygous loci 1388, 95, and 1024. The samples were tested with systems 1 and 2 at the same time. (A) The results for an individual without the c.1388G>A, c.95A>G, and c.1024C>T mutations. (B–D) The results for a homozygous or hemizygous individual with c.1388G>A, c.95A>G, and c.1024C>T mutations, respectively.

**Figure 5:**
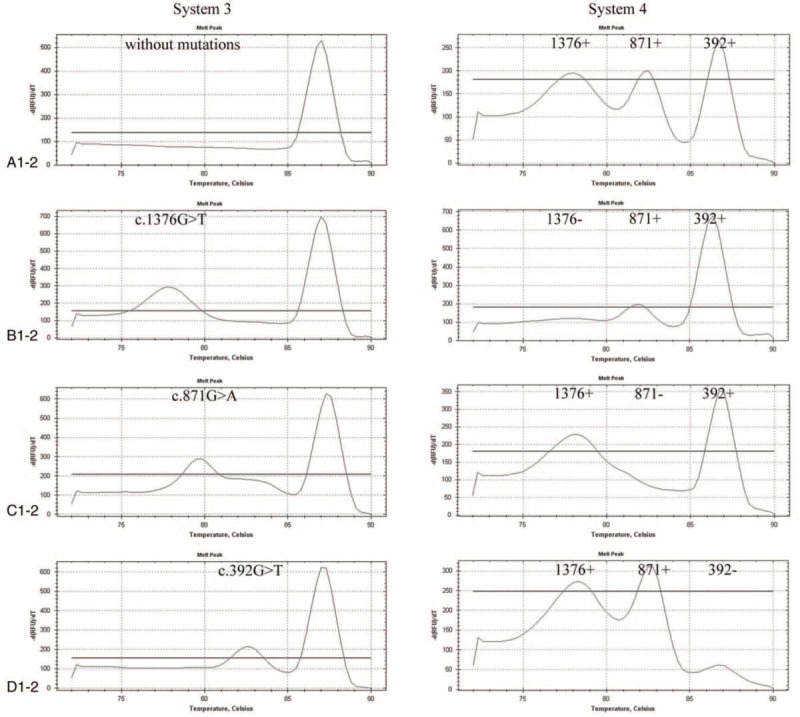
Melting curve analysis of homozygous or hemizygous loci 1376, 871, and 392. The samples were tested with systems 3 and 4 at the same time. (A) The results for an individual without c.1376G>T, c.871G>A, and c.392G>T mutation. (B–D) The results for a homozygous or hemizygous individual with c.1376G>T, c.871G>A, and c.392G>T mutation, respectively.

The results for a heterozygous individual with c.1388G>A, c.95A>G, and c.1024C>T mutations show in Figure 2. The results for a homozygous or hemizygous individual with c.1388G>A, c.95A>G, and c.1024C>T mutations show in Figure 4. The results for a heterozygous individual with c.1376G>T, c.871G>A, and c.392G>T mutations show in Figure 3. The results for a homozygous or hemizygous individual with c.1376G>T, c.871G>A, and c.392G>T mutations show in Figure 5.

Among the 174 cases of G6PD-deficient clinical samples that were evaluated by ARMS-MC, 1 or 2 of the 6 mutations were detected in 170 cases (including 5 cases of compound mutations); no mutation was detected in 4 cases. The number of individual mutations and the corresponding frequency of each are provided in Table 2.

**Table 2:**
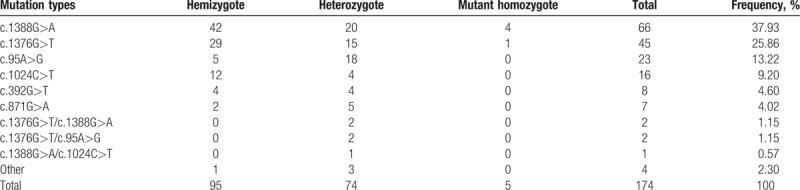
Genotypes revealed by genomic DNA validation tests.

The accuracy of the results for all of the clinical samples was verified by direct sequencing. All of the ARMS-MC detection results were consistent with the sequencing results. Sequencing analysis of the 4 samples in which no mutation was detected by the ARMS-MC method revealed 2 cases of c.1004C>T, 1 case of c.1360C>T, and 1 case of c.1311C>T/c.1360C>T.

## 4 Discussion

The ARMS-MC system established in this study can detect the 6 most common *G6PD* gene mutations in China. In addition, it can also distinguish the zygosity of mutations, including hemizygosity, heterozygosity, and homozygosity. The results of ARMS-MC analysis of the 174 neonatal G6PD-deficient clinical samples were consistent with the results of direct sequencing, demonstrating the accuracy and reliability of the system for genetic diagnosis. Among the 174 G6PD-deficient samples detected by the ARMS-MC method, mutations were detected in 170 cases; the c.1388G>A, c.1376G>T, and c.95A>G mutations were most common, accounting for 79.98% of all mutations. These results are consistent with those published by other scholars in China.^[12,14]^ The 6 detected mutations accounted for 97.76% of all of the identified mutations. This percentage is obviously higher than the results of Yan et al (85%),^[15]^ possibly because our samples consisted mainly of G6PD-deficient neonatal samples, including samples from more severely deficient cases. As a result, our samples likely included a relatively high proportion of mutations associated with severe G6PD deficiency. These results show that the ARMS-MC method we established can meet *G6PD* screening needs in the majority of the population in the region.

ARMS, which is also known as allele-specific amplification (ASA), can be used to screen for any known point mutation.^[16,17]^ However, due to its requirement of a high-quality primers, it is difficult to design an appropriate multiple ARMS detection system. Therefore, the design of the ARMS primers is key to the success of this experiment (shown in Table 1 and Fig. 1). First, the ARMS primers must amplify normal and mutation loci specifically. To ensure the specificity of the ARMS primers, we introduced bases that did not match the human DNA sequence into the primer sequence (the bold bases in the primer sequences) to prevent nonspecific amplification. Second, because each system needs to detect 3 or 4 different PCR products, primers must be designed that produce PCR amplification products of different lengths so that they can be distinguished by their *T*_m_ values. In addition, primer N95_up has 4 more bases than primer M95_up and introduces a base that does not match the DNA sequence. This design was used not only to increase the *T*_m_ value of the normal amplification product but also to avoid nonspecific amplification caused by high-temperature annealing. Furthermore, agarose gel electrophoresis analysis is required for traditional ARMS-based testing; this requirement makes the method more cumbersome to use. To develop the multiloci detection capability and the more convenient detection technology of ARMS, we combined ARMS technology with MC technology so that each system could simultaneously amplify and evaluate 3 normal or mutation loci. MC technology was used to distinguish and detect the amplification products because this method can not only reduce the inconvenience of the agarose electrophoresis detection but also avoid the possible contamination of PCR amplification products caused by opening tubes.

When designing the primer systems, we added endogenous controls (control 1 and control 2) to mutation detection systems 1 and 3, respectively. These controls were mainly used to judge whether a lack of amplification was caused by the absence of a mutation or the absence of DNA. In the normal locus detection system, the amplified products serve as endogenous controls for each other because homozygous mutations were never present at the 3 loci simultaneously. Therefore, no additional endogenous control was added. In addition, as loci 1376 and 1388 differ by only 12 bases, we designed a single common upstream amplification product that could simultaneously detect normal or mutated alleles at loci 1376 and 1388.

Although male and female samples were tested in the same ways, the focus of testing is different. Because there is only 1 X chromosome, without any corresponding allele, male samples are hemizygotes.^[18]^ Thus, the reaction systems 1 and 3 are sufficient to detect the presence of a mutation at one of the investigated loci. For female samples, as there are 2 X chromosomes, it is necessary to differentiate between homozygosity and heterozygosity. Thus, systems 2 and 4 must be used to detect normal alleles, whereas system 1 and system 3 are used to detect mutations.

Compared with the traditional ARMS, HRM, and dot-blot hybridization methods, the ARMS-MC method established in this experiment has the following advantages: single-tube multiloci detection has been achieved in this experiment; that is, a single tube can detect mutations at 3 loci, and 2 tubes can detect mutations at 6 loci. The detection efficiency is greatly improved. As the detection involves continuous PCR amplification and melting curve analysis, the PCR detection process takes only 2 hours to complete. A high degree of automation has been achieved for detection. Cumbersome hybridization steps are not required, which avoids contamination in the laboratory, and the machine automatically obtains the detection result. These advantages are even more obvious for large-sample screening.

## 5 Conclusion

The experiment results showed that ARMS-MC analysis could rapidly and conveniently detect the 6 most common *G6PD* mutations in Chinese people and distinguish hemizygous, heterozygous, and homozygous mutations. Therefore, this method can not only detect the common *G6PD* mutations but also provide a means to rapidly rule out these mutations when screening for rare or unknown mutations.

## Author contributions

**Conceptualization:** Zuqian Fan, Lei Sun.

**Data curation:** Guosheng Huang, Zhijian Pan, Weijun Tang.

**Formal analysis:** Lin Fang.

**Funding acquisition:** Lei Sun.

**Investigation:** Zuqian Fan, Tian Hu.

**Methodology:** Zuqian Fan, Xunjin Weng, zhao Long, Lei Sun.

**Project administration:** Lei Sun.

**Software:** Qiongying Fan.

**Validation:** Xunjin Weng, Yongxia Huang.

**Writing – original draft:** Lei Sun.

**Writing – review & editing:** Ju Long.
